# Clinical Reasoning About Timely Euthanasia of Compromised Pigs

**DOI:** 10.3390/vetsci12100943

**Published:** 2025-09-29

**Authors:** Julia Kschonek, Tristan Winkelmann, Lothar Kreienbrock, Elisabeth grosse Beilage

**Affiliations:** 1 Institute for Biometry, Epidemiology and Information Processing, University of Veterinary Medicine Hannover, 30559 Hannover, Germany; 2 Field Station for Epidemiology, University of Veterinary Medicine Hannover, 49456 Bakum, Germany

**Keywords:** timely euthanasia, content analysis, network analysis, reflection

## Abstract

In traditional livestock care, veterinarians often face situations where they have to determine the optimal time for euthanasia to prevent unnecessary suffering and pain. To improve timely euthanasia, this article focuses on the process of decision-making from a clinical reasoning perspective. The analysis aimed to identify typical steps of reasoning and explore the interdependencies between reported symptoms. For this means, decision-making reports from an online survey and in-depth interviews were assessed by an exploratory, sequential mixed-method content analysis. A key component of the approach involved generating a network analysis in Gephi to visualize the central symptoms associated with reasoning about euthanasia. The results also present a seven-step model of reasoning that can be used to innovate training concepts. Beyond practical applications, the findings have implications for theoretical considerations. Notably, the results suggest that euthanasia is more accurately conceptualized as a “script” rather than a simple “action”, highlighting the need for further exploration from the perspective of cognitive psychology.

## Simple Summary

When a disease or injury is incurable, caretakers or veterinarians have to decide when the right time for euthanasia has come. This decision can be challenging, and previous studies have primarily focused on influencing factors and the impact of training on the decision making. In contrast, this article shifts the focus from *what* knowledge is required to *how* knowledge is connected, thereby examining the decision-making process itself. The article generated a seven-step model of reasoning about euthanasia-worthy states of compromised pigs. Moreover, a network of symptoms was visualized, illustrating the key considerations involved in reasoning about euthanasia. This article serves as a foundation for trainers and veterinarians to enhance understanding about euthanasia from a clinical reasoning perspective. The main theoretical underpinnings are briefly discussed, while the findings are presented in a concise, visually oriented format to facilitate practical application.

## 1. General Introduction

The welfare of pigs on farms is a salient social issue. Especially when an individual pig suffers from diseases or injuries, it requires special care and attention to ensure the best chances for recovery. In some cases, however, curing is impossible, and caretakers or veterinarians have to decide when the right time for euthanasia has come to avoid unnecessary suffering and pain [1,2,3,4].

Finding the right time point, however, can be difficult in many respects. Previous studies have shown that challenges range from individual lack of knowledge and negative experiences to external factors such as economic concerns and regulation [1,5,6,7]. Based on the results, various efforts were made to address how the situation could be improved. In addition to training in euthanasia techniques, a focus was placed on imparting knowledge about diseases and guidelines to improve decision-making [3,8,9]. The success of the approaches, however, has been questioned [9]. It appears that neither imparting subject knowledge (about diseases and pathologies) nor the training of killing skills is sufficient to ensure timely euthanasia. One reason for this may be that what matters is not *what* is known and performed, but *how*. In other words, research has neglected to analyze the mere process of decision-making. Therefore, training approaches fail to train abilities such as synthesizing information from clinical examinations, defining diagnoses, interpreting results, and predicting chances. All of the mentioned aspects have been identified as central concerns to decision-makers regarding timely euthanasia [5]. Interestingly, all of these aspects refer to steps of clinical reasoning [10,11,12].

Clinical reasoning is a theoretical concept developed with the goal to enhance the understanding of expert knowledge and decision-making in medicine. It proposes that the knowledge about diseases acquired during university studies is organized in knowledge structures and refined over time through interaction with patients into “illness scripts”. The scripts summarize the most relevant, i.e., typical, disease characteristics in a list-like manner [13] (p. 614) and thus facilitate decision-making, which relied on unstructured networks before. In other words, illness scripts enable intuitive reasoning and quick judgment based on limited information. In contrast, analytical reasoning requires time, global information, and stepwise deductive logic of thought to derive the correct diagnosis and prognosis from patient encounters [12,13,14,15].

The previous paragraph merely summarizes the basic pillars of the analytical concept, which has been researched for decades. One key result of the investigations for this article is that gained knowledge about the analytical processes was recently transformed into veterinary training concepts [10,11]. According to these concepts, the logic of reasoning can be improved by conveying knowledge about the eight typical steps of clinical reasoning and analyzing a clinical case study or patient encounter using these steps [10,11,16].

The goal of this article is to use the central assumptions of these clinical reasoning concepts to enhance the understanding of euthanasia decision processes. Euthanasia is a potential endpoint of many illness scripts, i.e., mental knowledge structures that store the most relevant information about a disease and, once enacted, lead to further action. Because euthanasia is a potential endpoint of many illness scripts, we assume that experts possess a euthanasia script that symbolizes a condition that unequivocally gives rise to euthanasia (i.e., slots of illness scripts in theoretical terms [14] (p. 1179); to facilitate reading for practitioners, we refer to scripts at this point). While it is beyond the scope of the article to test this hypothesis, this article will explore which typical steps and symptom networks (knowledge structures) can be defined with the help of clinical reasoning concepts. For this goal, an exploratory convergent mixed-method analysis was performed to assess reported decision processes in an online survey and in-depth interviews. Details of the methodology are further defined in the Section 4. The Section 2 summarizes the knowledge about clinical reasoning concepts and euthanasia of compromised pigs that is necessary to understand the results and develop training concepts based on the findings of this article.

## 2. Conceptual Background

### 2.1. Euthanasia of Compromised Pigs

The decision to euthanize a compromised pig becomes necessary when a pig suffers from conditions that cannot be treated or/and are incompatible with life [4,17]. Sometimes, the term “euthanasia” stands for the killing of pigs by veterinarians with the help of medicine. Otherwise, the terms “killing” or “emergency killing” are used to describe situations when compromised pigs are killed on the farm for other reasons than slaughter [3]. The article refers to the term “euthanasia” as the timely relief of a pig from a condition that cannot be treated or/and is incompatible with life. This is performed by both veterinarians, farmers, and caretakers, as they frequently decide on and perform the euthanasia of pigs and therefore need to know and identify relevant disorders.

The process from identifying relevant disorders in compromised pigs to conducting euthanasia is not yet well researched. More specifically, studies have shown that a multitude of farm, economic, and personal considerations influence decision-making processes [2,3,5,6,7,10]. However, the exact steps in the reasoning process are still unclear. Results in studies suggest that timely euthanasia depends on an early identification of affected animals [2,5]. Moreover, the processes of interpreting clinical results and predicting chances of recovery pose a challenge for decision-makers. Summarizing, there is a great interest in strengthening reasoning skills, i.e., ensuring that considerations are good enough to support one’s decision on how to proceed [5].

When asking veterinarians and farmers about common reasons for euthanasia of compromised pigs, answers depend on the animal’s age and subsumed low viability, broken bones, non-ambulatory states, and lameness, among others [5]. It should be emphasized here that terms like “non-ambulatory” represent symptoms from a semantic point of view, whereas the mentioning of a medical/etiological diagnosis was expected [5]. Certain symptoms thus seem to play an essential role in reasoning about euthanasia and need more attention in research. This point can be further supported by the finding that certain symptom categories, such as wound severity, signs of generalization, and impact on gait or food and water intake, were common and independent of the diagnosis in a recent review [2]. In this respect, the often mentioned interest to enhance understanding of valid symptoms for good reasoning may be explained in an online survey about influences on euthanasia decision processes [5]. Elaborating on the question of which symptoms represent euthanasia-worthy states of compromised pigs, and more specifically, how the symptoms interdepend throughout the reasoning, is thus an important research topic to be addressed in this article.

### 2.2. Clinical Reasoning

Clinical reasoning is a middle-range descriptive theory that was generated with the goal to analyze expert knowledge and, conversely, to improve the decision-making of novices in medicine [12,13]. Research has been conducted about clinical reasoning and its practical applications for decades. The term is to be understood as an umbrella for propositions about making meaning, decisions, and the associated skills. Due to this, there is not one concept of clinical reasoning. Rather, the focus of analysis and training approaches can be tailored to a domain perspective [12,18,19].

The paper focuses on clinical reasoning in veterinary medicine with the goal to analyze decision processes and symptom networks reported by experts in interviews. A recently published veterinary training concept will be used for orientation. In this concept, clinical reasoning is depicted as a logical process which includes the collection and processing of information as well as the planning and reflecting on actions taken [10,11,16]. Further core propositions are presented in the following section.

#### 2.2.1. Assessing Clinical Reasoning

**Figure 1:**
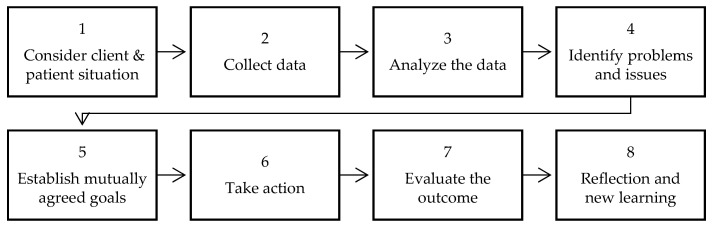
Steps to assess clinical reasoning in [10].

To facilitate understanding and training of how to make meaning and decisions in medicine, the process of clinical reasoning is separated into eight steps (Figure 1).

Seven of the eight steps describe the process of a clinical encounter and thinking. The last step serves to reflect on the process and to learn about decisions that have been made during the following process (oriented on [10,16,20]).

A clinical encounter generally starts with considering the client’s and patient’s situation (1). This includes gathering information such as the name of the patient and the patient’s context or persons involved in the process. Subsequently, veterinarians start collecting data (2) by help of a health interview, clinical examination, and ancillary techniques such as collecting samples for bacterial culturing. Based on the data, veterinarians (to facilitate reading, we refer to veterinarians in the description despite that our results include and address farmers as well) process the information and analyze data (3) to identify the problems (4) and issues of the case. For steps three and four, several distinct activities can be defined as outlined in Table 1.

Once the problem has been defined, veterinarians seek to establish mutually agreed goals (5) with the client and consider taking actions (6) that range from treating to separating, to performing ancillary tests. Finally, veterinarians evaluate the outcome (7) to assess how actions worked out and whether the treatment protocol has to be adapted for the patient.

The last step of the clinical reasoning process is to reflect and learn (8) for future clinical encounters. In a training situation, the activities and skills needed for a reflection (Table 2) may need supervision.

With the help of the seven steps and reflection about a clinical encounter, veterinarians shall acquire clinical reasoning competencies and awareness about their weaknesses during the process [10] (p. 19). Moreover, the steps serve to guide the analysis of decision processes. Some additional theoretical concerns need to be addressed regarding the logics of thought and scripts used for recalling knowledge.

#### 2.2.2. Logics of Thought in Clinical Reasoning

The interest in clinical reasoning relies partly on the assumption that illness scripts enable correct and efficient decision-making about a case [14]. The moment a patient is seen, a veterinarian decides on the further course of action. The logic of thought behind this process, inductive reasoning, describes that inferences are drawn without cognitive effort from the long-term memory [13,14,18]. Synonyms of this logic refer to judging upon gut feeling, intuitive type of reasoning, or quick judgment based on tacit knowledge (also see the Glossary in [10]).

The organization of enacted knowledge is referred to as illness scripts (further explained in the next section). They mature through experience and patient encounters over time, which means that repeatedly encountered information, cues, and symptoms generate a dominant cognitive path, or “slot”, of a script. As soon as this information is perceived, it activates a particular (slot of a) script and supports quick judgment [14,18]. Experts had enough time and patient encounters for the mind to generate scripted, i.e., shortcut cognition paths. In contrast, novices in medicine consider every potential connection of biomedical knowledge during a patient encounter. Their reasoning is that conscious stepwise needs additional information and time. The corresponding logic of thought is deductive and often called the analytical type of reasoning [13,14].

When the concept of clinical reasoning was under construction, discussions were made about whether the analytic or intuitive logic of thought is predominant for analyzing reasoning processes in medicine. Nowadays, it is assumed that the two types of reasoning interact (called the dual type of clinical reasoning). The primary logic of thought depends on the patient information and individual long-term memory scripts (among other factors) [11,13,19]. (Additional information about cognitive theories and assumptions and logics of thought are documented in [12,18,19]). Since this article aims at generating a first exploration of clinical reasoning logics in euthanasia decision-making, the definitions presented above are enough to assess whether one of the logics is recognizable or whether a shift can be observed when analyzing reported decision processes. The most important theoretical foundations of an illness script are further defined in the following.

#### 2.2.3. Scripts in Clinical Reasoning

Summarizing a review on illness scripts [18], a script defines a high level structure in the mind that organizes information to facilitate understanding of behavioral (or causal) sequences of real-life events. When encountering stereotypical situations (such as a patient encounter), involved persons have tacit knowledge of what is going on and how to behave and—given the information—can predict correctly how the situation will proceed. Information in the scripts is generated through learning and experience and is thus flexible. There is neither *one* script, nor is information exclusively linked to one script. Yet, information gain attributes to be typical (central) or atypical (peripheral) to grasp the situation [13,14,18].

For means of illustration, from the whole dense network of medical knowledge and information about pig diseases (stored in the long-term memory), an illness script for dealing with a suspected infection of *Streptococcus (S.) suis* will be enacted when a veterinarian encounters a suckling pig that is non-ambulatory, paddling, and exhibits convulsions, as the presented symptoms, age, and context are typical of the situation. Of course, an alternative diagnosis may be valid, as convulsion may be a sign of any other neurological disease ([21], (p.68ff.)). Similarly, a pig may be non-ambulatory due to a lameness (i.e., the symptom would belong to a lameness script). Throughout the reasoning process, however, a veterinarian will intuitively or deductively collect cues to reach a sufficient match of the patient’s information with those of a specific illness script to decide about the further course of actions.

Categories that are commonly considered to structure illness scripts are enabling conditions, such as predisposing factors (e.g., hereditary and genetic influences) or boundary conditions (e.g., sex or age, such as suckling pigs are predisposed for *S. suis* infections), the fault (e.g., pathogenic organisms such as *S. suis* are common pathogens for CNS infections), and consequences (e.g., signs and symptoms of the patient such as convulsions) [13,22]. The three categories are neither consciously created nor the only categories used for structuring an illness script. For this article, however, the categories can be used for analyzing reported scripts in the decision processes about euthanasia.

#### 2.2.4. Euthanasia in Clinical Reasoning

The above discussed eight steps and theoretical considerations of clinical reasoning can be used to analyze or to train reasoning about a clinical case. In the study of Agne et al. [10], the mastitis of cows is used as a typical bovine disorder for training and analysis. In this case, euthanasia is considered as one potential action that is either supported or rejected on the basis of reasoning processes.

As discussed in the previous section, an illness script provides the rationale for determining the course of action for a patient. We assume that there is at least one slot in the illness script that contains typical information sufficient to justify or rule out euthanasia. This slot may be part of a general illness script, or, given that euthanasia is a potential endpoint for many disorders, it may be a unique script specifically designed for euthanasia-worthy states.

To illustrate this concept, consider a pig suffering from disease X (such as S. suis infection). If the pig displays information that is typical and indicative of euthanasia, this information may be specific to the endpoint of a particular disorder (e.g., convulsions associated with S. suis infection) or it may be shared across multiple disorders (e.g., refusal to eat or drink after either S. suis infection or severe lameness). In either case, the information would be characteristic of a euthanasia-worthy state script.

While the considerations cannot be tested in this article, the analysis will show whether and which symptoms are central to reasoning about euthanasia, whether they are exclusively tied to particular diseases, and whether steps of reasoning need to be adapted to represent reasoning about euthanasia of a compromised pig.

## 3. Materials and Methods

The goal of the article is to explore how the perspective of clinical reasoning enhances understanding of decision-making processes about euthanasia of a compromised pig. In particular, the questions examined are whether typical steps of clinical reasoning can be identified throughout decision-making and how reported symptoms interdepend throughout reasoning.

The data stems from an exploratory, sequential mixed method approach. Data collection started in October 2022 and ended in June 2023. One dataset refers to an online survey with *n* = 72 open responses of veterinarians (*n* = 28) and farmers (*n* = 44). All participants have worked on average more than 5 years with pigs and conducted their last euthanasia in the past 2 weeks (75% farmers, 78% veterinarians). They were asked, “How do you decide whether a compromised pig needs to be euthanized? Note: Formulate a decision-making process in key words”. The focus was not limited to a certain disorder or injury. Answers to this question were not published before. Other results of the online survey are published in [5].

The second dataset stems from in-depth, structured expert interviews of three veterinarians and four farmers. The participants have worked with pigs on average for about 20 years (range 9–32 years), and they conducted their last euthanasia within the last week (except for one missing value and one person who conducted euthanasia about a year and a half ago). The questions asked about typical disorders for euthanasia as a first step. Related to the disorder, interviewees were asked how they identify the animal at first and what indicates that the disease develops towards recovery or euthanasia. The exact questionnaire is provided in Appendix A. The length of interviews varied between 30 and 75 min.

Data collection was approved by the data privacy officer of the university. Data was gathered only in cases where informed consent was granted prior to the interview/online survey. (See details about the ethics in the informed consent statement).

The analysis of data (oriented on [23,24,25,26]) proceeded in four sequential, convergent steps. Firstly, the interviews were transcribed and inductively coded based on the research question and prior knowledge of the author. Secondly, the free texts from the online survey were analyzed deductively with the help of the eight steps of clinical reasoning and theoretical considerations discussed in the Section 2. Thirdly, results of both analyses were compared and used to extend or refine the fit of codes (conversion). To validate and increase the quality of the revised set of codes, the fourth step led to recoding the datasets with the revised coding scheme again.

While the revised codebook can be reuse on request of the author, some methodological considerations need to be emphasized. Generally, all reported information is from the participant’s memory. In theoretical terms, every piece of information might be a cue in a script for evaluating a disorder or a euthanasia-worthy state [14,24]. Some of the questions were directive (e.g., “What of this pig attracts your attention on day one”, which suggests a first momentum of identification), wherefore not all parts of the interviews could be used for all analysis questions.

Reasoning is rather a cyclical than a linear process [12,16]. Phrases of a text can therefore represent more than one code at a time (e.g., “re-assessing treatment” indicates that “treatment” is a potential action (step 6), and “re-assessing” shows that the outcome is evaluated (step 7)), especially when assessing codes of symptoms that can indicate what is performed throughout clinical examinations (step 2, data collection), what symptom is considered relevant (step 3, interpretation), and potentially how symptoms interdepend (step 4, matching). In the Section 4, a reference to the coded (raw) text is provided where substantial inferences are made in the form of “(Person: Line of reference)”. If one coded text is referenced, it will be placed in the text. If more coded parts are referenced, they are placed in a separate box.

Focusing on the relation of symptoms, a final step of the analysis was to generate and visualize a network of symptoms depicting euthanasia-worthy states. For this means, coded text was transformed into a source (starting point of argumentation) and target (end of argumentation); for example, a broken bone (source) causes pain (target). The direction could have a negative, positive, or neutral connotation.

The source-target-direction code was imported to the network visualization tool Gephi (Open Source Network Visualization Tool, Version 0.10.0, see [27,28]). In Gephi, the sources and targets were visualized (as nodes) and connected (as edges). Based on the count frequency of a node in the data and its number of edges, the weight and proximity of nodes were visualized by help of the algorithms “Circular Layout” and “ForceAtlas2”. While the “Circular Layout” basically organizes nodes according to weight or attributes defined in the dataset, “ForceAtlas2” is a continuous algorithm which translates structural into visual proximities [29] (p. 2). In principle, the algorithm serves to repulse nodes while edges attract their nodes (i.e., nodes are placed depending on other nodes) [29] (p. 2). The visualization is scale free and not deterministic. This helps to avoid imposing structural assumptions on a network by generating different layouts [29] (p. 2) and supports the aim of this article to explore symptom networks in reasoning about euthanasia decision-making. The CSV-formatted sheets for generating a network visualization in Gephi are available in English for reuse on request of the author (see Data Availability Statement).

## 4. Results

The results begin by describing clinical reasoning about euthanasia of a compromised pig as a stepwise process. Subsequently, detected logics of thoughts will be reported.

### 4.1. Steps in the Process

The analysis of euthanasia decision-making processes led to a decision-making model with seven steps and the associated activities (Table 3).

The steps will be further described and illustrated in the following sub-sections.

#### 4.1.1. Step 1: Identification of Animals

The identification of a compromised pig is the first step of euthanasia decision-making. It is important to **identify a pig early** (1A) and to reassess both potential and identified compromised pigs up to several times a day. The early identification is rather defined as a process of intentionally looking for a compromised pig instead of finding it occasionally in the herd during a daily inspection (Box 1).

Box 1 **Identifying animals**

“You have to secure early identification” ^(D:28)^

“Recognition, marking and separation of the animal” ^(L35:1)^

“But you have to recognize the clinical picture first, and that is difficult enough” ^(TC:49)^

“Sometimes you find them in a herd of 50 other pigs” ^(TA:63)^

#### 4.1.2. Step 2: Gathering Information/Data Collection

The second step of euthanasia decision-making is to collect information about the compromised pig and the farm. The data collection is represented by **specific actions** (step 2A), the definition of **relevant outcomes** (2B), and considering **patient circumstances** (2C).

**Specific actions** (2A) refer to the literal conduct of clinical examinations but also the use of mnemonics, for example, when assessing inflammation (“Is it warm, swollen, reddened?” TA:55). Farmers and veterinarians repeatedly emphasize the targeted search for compromised pigs or signs of a health problem, walking around the pens, and marking the animals to allow for a reassessment of their condition and that of other animals. Less frequently mentioned are the following measures: touching compromised pigs (e.g., the joints to detect signs of inflammation), taking measurements (e.g., to detect fever), or listening for signs of pain such as squeaking. More frequently mentioned is the attempt to encourage a compromised pig to stand up when it lies on the floor.

**Relevant outcomes** (2B) that data collection should generate include patient data (2B1) such as the class of a pig. It is represented by the age and weight (e.g., a day old suckling pig; a 400 kg breeding sow (note of the authors: the weight of the sow was actually that high, although sows tend to weigh less)), the stage or use of a pig (e.g., rearing department, use for fattening or reproduction means), and its genetic background (e.g., Danish breed). Furthermore, the locality of the pig is frequently assessed regarding conditions of food and water supply or whether it is separated in a hospital pen. In addition to this patient data, clinical examination goals (2B2) and/or abnormal symptoms (2B3) are described: when examining pigs, farmers and veterinarians look for signs of “normal behavior and performance”. This category of signs includes the activity, sensorium, and interaction of a pig. When a pig behaves differently than its pen mates, i.e., lies separately, avoids interactions, shows teeth grinding or squeaking, it shows abnormal symptoms. Beyond signs of behavior, pigs are examined for signs of “abnormal posture”. This category of signs includes the ability to stand, sit, and lie normally and, conversely, a non-ambulatory state or hunched back. In addition to that, the “capability to walk” is assessed. It is impaired when the load of a limb is reduced, the motivation to move is low, joints are enlarged and inflamed, or a leg bone is broken. The “capability of eating and drinking” and body condition of a pig are examined as well. Related abnormal symptoms include no intake of food or water, the loss of weight, the spine sticking out, or the flanks being sucked in. Concerning the digestive tract, defecation is mentioned as another point to assess. Furthermore, signs of inflammation or infection are assessed, such as warmth, swelling, and redness of the skin. When the “integument” is assessed, veterinarians and farmers look for injuries and perforations. Finally, the “changes over time” are considered regarding examination results or treatment of the previous day” (e.g., whether the swelling is smaller, whether the protective posture is still taken, or whether signs indicate a chronic process versus an acute exacerbation). In most reported processes, participants stated the goal to identify injuries and disorders specifically instead of identifying anatomical abnormalities. The goal to detect pain and suffering was mentioned as well, a point that will be referred to later again.

A final goal of the data collection is to consider **patient circumstances** (2C). The inferiority of a compromised pig was specifically assessed and describes whether the pig is being run over by his pen mates, or whether it has lower chances to compete for food and water.

#### 4.1.3. Step 3: Analyzing and Interpreting Data

The data from clinical examinations is analyzed and interpreted in a third step of euthanasia decision-making. This article begins reporting **suspected hypotheses** (3A) of a case and proceeds with **clinical** (3B) and **contextual arguments** (3C) for the hypotheses. Finally, findings elaborating on the **recalled scripts** (3D) will be summarized. (This approach (beginning with suspected diagnoses) facilitates reading, although we would assume that a hypothesis is the final result of a reasoning process.)

Defining a clear suspected diagnosis is outlined as an important activity when evaluating euthanasia. The **suspected hypotheses** (3A) about the health state of a pig can be grouped into those mentioning specific diseases/disorders and those evaluating euthanasia-worthy states (3A1). Specific diseases refer to injuries of the integument or inner organs, prolapses of the rectum, lameness, tail-biting, reproduction disorders, infection with *S. suis*, impaired joints, umbilical outpouchings, and broken bones, among others. In about twice as many reports, when asked how to proceed in evaluating euthanasia, no particular disease or disorder is mentioned, but a list of arguments and symptoms is. These responses are considered to evaluate the hypothesis of a euthanasia-worthy state regardless of any specific diagnosis or disorder.

The specificity of a hypotheses (3A2) is part of reasoning processes as well. If a clear diagnosis is given, the certainty of deciding on treatment is raised: “It is important that pigs have a clear disorder that we can treat, otherwise, well, you see the pig is behind and shows these symptoms—well, what can you do”, (TB:58). The association of a clinical case with a clear diagnosis is also relevant to impart knowledge: “We can train people, especially when it is a clear case”, (TB:32). Yet, the etiological diagnosis is not sufficient as a sole reason when it is outlined: “[Decision-making] is very difficult for the Streptococci-cases”, (A:63). Interestingly, defining the specific disorder also does not seem to make a difference in some cases: “I don’t know why the sow is non-ambulatory. Well, the veterinarians would have to make a full clinical examination. Usually, this is not done. If the sow doesn’t get up, there is nothing you can do”, (D:33–42). Summarizing, a diagnosis that can be specified seems to solidify considerations about further actions. If the specificity of a diagnosis is low, however, the focus shifts to specific symptoms to develop a hypothesis about the pigs’ health status.

To support a suspected hypothesis, interviewees made several **clinical arguments** (3B). The category of matching (3B1) summarizes arguments when participants associate the identified symptoms with medical terms: “Well, I see a sow, she is tiptoeing, so I would say: lameness-degree I”, (TA:49). The category of inferring (3B2) describes how interviewees make inferences from seen symptoms to other information: “When they cannot stand, they cannot suckle”, (A:59). Many arguments refer to the category of relating (3B3), in which the current state of the pig is interpreted with regard to animal welfare, pain, suffering, or the overall state of the pig (Box 2).

Box 2 **Relating Clinical Symptoms**

“When my peers or I personally feel that the animal is suffering” ^(C:44)^

“When pain is obvious” ^(25:1)^

“I would say, it is the overall appearance of the pig” ^(A:72)^

Participants are also predicting (3B4) the further course of action. Coming to a prognosis is hereby mentioned both literally and descriptively. In descriptive terms, participants argue in favor of euthanasia because this is how a disease usually progresses. In a similar vein, the likelihood of the suspected diagnosis is made explicit by outlining alternative hypotheses (Box 3).

Box 3 **Predicting the Course of Action**

“That’s always the same story in the rearing site” ^(TA:43)^

“It can be, but I am not a 100% sure” ^(D:38)^

“Well, you have to consider: is this just a small animal, is it infected, did it just have a bad week- and whether it may come to better terms.” ^(B:44)^

When predicting the further course, another endpoint is the likelihood of the compromised pig to become a slaughter pig. More precisely, a high chance of failing to become a slaughter pig is a strong argument to decide in favor for euthanasia (Box 4).

Box 4 **Likelihood to Become a Slaughter Pig**

“Is it fully marketable after curing?” ^(42:1)^

“Well, you wouldn’t wait much longer how the animal develops, because it is quite clear already that the pig won’t be slaughtered, won’t reach its target weight” ^(TB:56)^

Predicting the chance to recover is a major focus of considerations, too. Curing is described as an argument against euthanasia (restitution ad integrum), and, conversely, no prospect of recovery as an argument supporting euthanasia. Furthermore, curing with a remaining defect (restitutio cum defectu) and functional curing (ad functionem) are described as well (Box 5).

Box 5 **The Chance for Curing**

“The chance of curing is always considered” ^(A:77)^

“No prospect of curing” ^(F11:1)^

“The swelling can become a hardening, but as long as they are able to walk…” ^(A:77)^

“Treatment is successful first, then the situation worsens again—this leads to euthanasia” ^(T20:1)^

Curing is often evaluated considering the possible treatment options. Elaborating on the worthiness of treatment is another major argument when evaluating euthanasia decisions (Box 6).

Box 6 **Worthiness of Treatment**

“Antibiotics cannot resolve such a big pus spot” ^(A:77)^

“Is it worth treating?” ^(L27:1)^

“Either treat or euthanize. When treated and no success afterwards, euthanize. Otherwise go on with the treatment.” ^(L39:1)^

Apart from predicting the further course of a disease, the displayed symptoms of a pig are related to other information. Discriminating (3B5) arguments describe how symptoms are assessed regarding inconsistencies (e.g., the pig stops eating but has no fever; A:38). Or symptoms are evaluated as signs of generalization (e.g., if a second joint is affected, the infection has generalized; A:74). In many cases, however, the significance of a symptom is specified based on its duration. The symptom “non-ambulatory”,defined as a situation when a pig is unable to retain any other position than lying, for example, is acceptable for 24 h but not for several days. This argument was also used for the signs of “taking up no food and water”, “suffering”, and “pain”. For the symptoms of “broken bones” and “generalization”, immediate euthanasia is considered.

**Figure 2:**
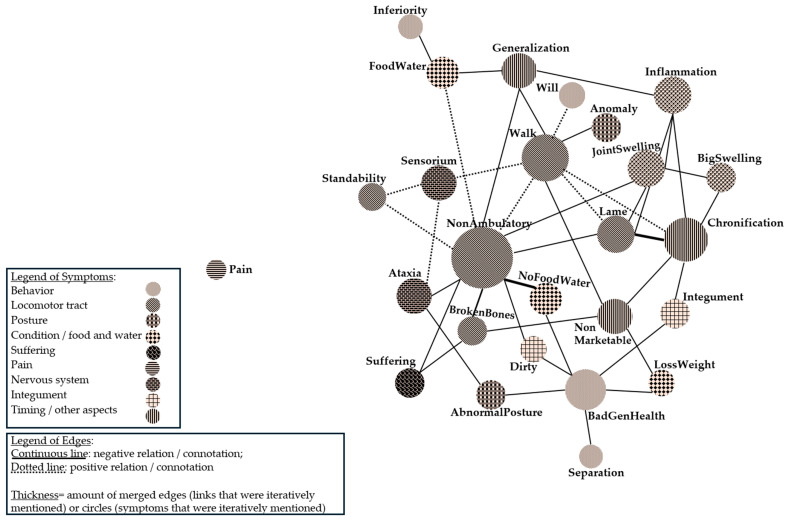
Network of symptoms. The position of the circle “Pain” was manipulated by authors to fit the image onto one page. To make the presentation accessible, the graphics were re-colored by hand.

To better understand the relationship between the symptoms, a descriptive network analysis was conducted using a visualization tool. In a first step, the symptoms were presented in the context of the disease/disorder with which they were named. In total, 67 symptoms were mentioned in relation to the following diseases/disorders: infection with *S. suis* (“FallOverPigs”), accidents, tail biting, joint disorder, low viable pigs, locomotor diseases, umbilical hernia, the mentioning of suffering and pain, and miscellaneous (other). In this step, one symptom was unique for the disorder of “umbilical hernia” (i.e., perforation of umbilical hernia) (the authors emphasize that perforation usually occurs after long-standing ulcerative skin lesion), and three symptoms appeared to be unique for the disorder of “low viable pigs” (i.e., inactivity, good general health, and scrubby skin). Due to the connectivity of all other symptoms, a second analysis was performed to visualize the symptoms independently of the original disease, but with regard to the association of symptoms. The result is visualized in Figure 2.

The network in Figure 2 visualizes symptoms (in circles) that were mentioned in relation to another symptom in decision reports. The links do not impose a causal inference. A relationship of symptoms is either in a negative context (solid line, e.g., when pigs cannot stand, they cannot consume food and water) or in a positive context (dotted line, e.g., when a pig can walk that was non-ambulatory before). For the illustration, similar links were merged (the number was added), which is represented by the thickness of a link. As a result, more frequently mentioned links appear thicker, and less frequently mentioned links appear thinner in Figure 2. Similarly, a frequently mentioned symptom appears as a bigger circle than a less frequently mentioned one. To structure the network, the symptoms that are linked are positioned closely, and more frequently connected symptoms are closer than other less frequently connected symptoms. Hence, the sizes of circles and lines, as well as the approximation of circles, serve to express the structural relation of symptoms.

Figure 2 visualizes that symptoms of the locomotor tract (horizontal lines) and particularly the symptom of a “non-ambulatory state” are of central importance for reasoning about euthanasia. The transition from a “non-ambulatory state” to the “capability to stand or walk” defines a positive connection. Conversely, the connections of “ataxia” and “broken bones” with a non-ambulatory state represent negative contexts. “Non-ambulatory states” are often named in relation to the “inability to take of food and water” (dotted grid: “NoFoodWater”). Mirroring “non-ambulatory states” is a symptom of “ability to walk” (top left to bottom right lines: “Walk”). It is positioned in relation to the motivation of the pig to live (vertical dotted grid: “Will”), and defines a positive transition from signs of impaired locomotion such as lameness (top left to bottom right line: “Lame”). A frequently associated sign of locomotor symptoms is “Chronification”, standing for a prolonged or exacerbated compromised state. Another typical clinical sign when evaluating euthanasia is the impression that the pig is in a “bad general health status” (vertical dotted grid: “BadGenHealth”). It is positioned in relation to impaired and dirty skin (rectangle pattern: “Integument”), bad condition (check pattern: “LossWeight”), and abnormal behavior such as separated lying (vertical dotted grid: “Separation”).

**Figure 3:**
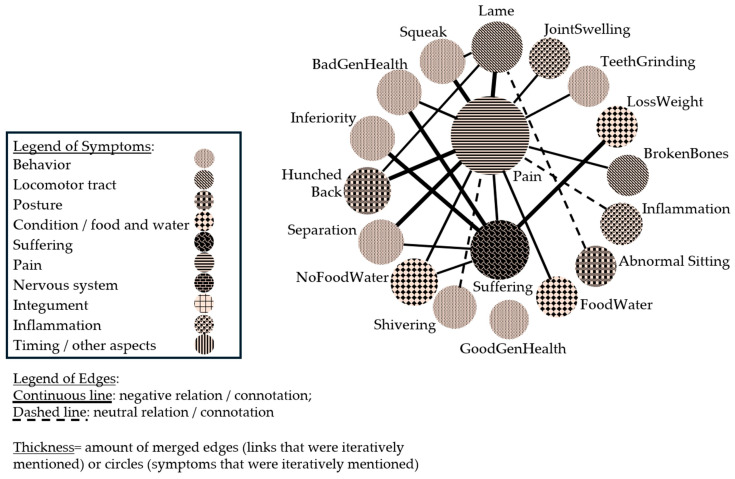
Network of symptoms focusing on pain and suffering as reported by interviewees. To make the presentation accessible, the graphics were re-colored by hand.

The presentation of suffering and pain needs to be addressed as well. Suffering is mentioned in three reports in relation to the symptoms of “non-ambulatory states”, “broken bones”, or “inability to take up water and food”. Pain, in contrast, is mentioned in four reports but not in relation to any specific symptom. Since participants self-reported the aim of detecting pain and suffering of a pig, the identification of both symptoms was specifically addressed in the interviews. Interviewees were asked how they identify signs of pain and suffering in a pig. Responses are depicted in Figure 3.

Figure 3 visualizes symptoms in relation to pain and suffering from the interview partners. Similarly to previous figures, the size of the circles and edges represents the frequency of counts in the data. Figure 3 visualizes that more symptoms were named to describe the identification of pain compared to suffering. The major symptoms related to pain are lameness (“Lame”), squeaking (“Squeak”), a hunched back (“HunchedBack”), and no uptake of food and water (“NoFoodWater”). While pain is rather defined as the cause for abnormal symptoms, suffering is rather described as the result of abnormal symptoms. Exclusively linked to suffering are “Inferiority” (competition with pen mates) and the loss of weight (“LossWeight”).

**Figure 4:**
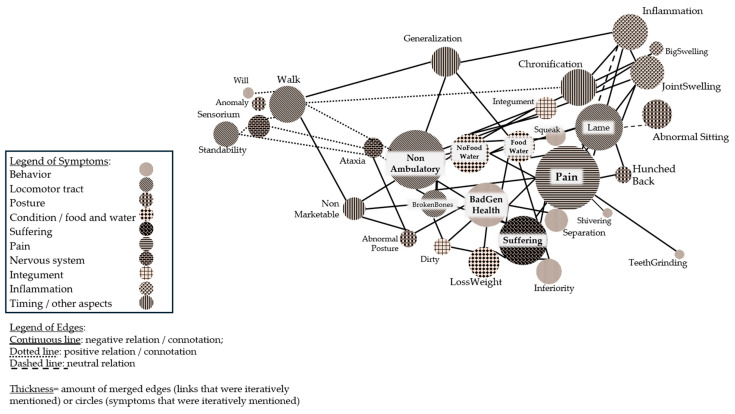
Network of symptoms, including elaborations on pain and suffering. The position of “hunched back” was manipulated by authors to facilitate reading. To make the presentation accessible, the graphics were re-colored by hand.

A comparison of the symptoms associated with pain and suffering with those mentioned when evaluating euthanasia in general (Figure 3) shows that the symptoms of teeth grinding, shivering, squeaking, abnormal sitting, and hunched back are only mentioned when asking about signs of pain or suffering. The other nine symptoms are mentioned in relation to particular diseases or disorders as well. When joining the interview reports about pain and suffering with online survey reports, the analysis shows that pain and suffering gain a more central position for reasoning about euthanasia-worthy states (Figure 4).

Figure 4 shows results of reports where interviewees were explicitly asked about pain and suffering. Several changes in the network can be traced: on the one hand, the circle size of pain increased and now resembles the circle of non-ambulatory states. The circle of suffering changed from a small circle with a peripheral position to a central position closely associated with bad general health. The circles for general bad health, lameness, and inflammation furthermore increased in size, while the size of “Chronification” decreased. Moreover, a cluster on the left side of Figure 4 is separated, showing the ability to stand (“Standability”) and walk, among others. This cluster represents clinical signs indicating recovery.

To facilitate later discussion, a summary of the figures is provided. First, it is evident that symptoms are viewed less in relation to a specific illness than to other symptoms. Central positions are taken by signs of the locomotor system, such as a non-ambulatory state, but also by behavioral characteristics. Although pain and suffering are rarely mentioned, they are central to the symptom network when asked for their identification. Furthermore, including considerations about suffering and pain improves the network structure, as clusters are formed that separate clinical signs for recovery.

This next paragraph shifts the focus from symptoms of a pig to **considerations of the context** (3C) that influence decision-making about euthanasia. A first set of arguments describes the characteristics of involved persons (3C1). Considerations include whether a person is challenged by the discomforting nature of the topic. Results suggest that involved persons need to be able to identify disorders, but also to feel empathy for the pigs. Moreover, decision-makers need to be accountable and concentrated when looking for a compromised pig. They need to feel in charge to make decisions and conduct subsequent actions. A second set of arguments describes how institutional structures (3C2) shape decision-making, such as economic rules and regulations for transport and slaughter. External or public control is often mentioned, which refers to sanctions for decisions that are not well-founded. A third set of arguments summarizes considerations about the farm and local equipment (3C3). Techniques available for conducting euthanasia are of concern, but also the frequency of veterinarians’ visits or treatment documentation. Lastly, an “individual pigs” theme needs to be mentioned. It stands for the repeated argument that this case, i.e., the pig we are talking about, is just one single case, and it differs from all previous experiences: “I have killed no single animal for this reason”, (B:35).

A final aspect to be assessed in the reports is signs for **recalled scripts** (3D). In the online survey reports, it is literally mentioned that decisions depend on experiences with comparable clinical pictures. Otherwise, signs to illustrate specific illness scripts (3D1), such as enabling conditions, the fault, and consequences, can be seen in the example of “tail biting”. In order to explain decisions about the further course of a tail-bitten pig, the pathology of the disorder (i.e., the infection has affected the spine) and consequence (i.e., the animal is non-ambulatory) is mentioned for decision-making (L5:1). Similarly, cases of joint disorders were described regarding fattening pigs (enabling condition) in which the infection of the joint is suspected (fault) and results in impaired gait/protective posture (consequence).

Semantic qualifiers (3D2) can be used to analyze whether a script is enacted. (They are opposing pairs of adjectives or adverbs that qualify a symptom to enact a certain script. Examples are acute vs. chronic, dull vs. sharp, frequent vs. rare [10].) In the analysis of interview reports, qualifiers with respect to considerations of the likelihood of a disease to occur (frequent vs. seldom) and the count of affected body parts of a pig are mentioned. Moreover, a symptom is often described with the help of the terms “low-high” (e.g., lameness grade), “slight-severe” (e.g., pain), “moderate-extreme” (e.g., pain). Finally, the size of an affected body part or the degree of an open wound is used as a semantic qualifier.

#### 4.1.4. Step 4: Mutual Goals

The definition of mutual goals in decision-making is the fourth step of euthanasia decision-making. Once a hypothesis about the state of the compromised pig is elaborated, veterinarians and farmers consider which mutual goals they need to take into account. One set of goals addresses the **population health consequences** (4A), such as whether a compromised pig is a risk factor for the herd. A second objective is to ensure effective **consultation and communication** (4B) between veterinarians and farmers. Trust and respect are mentioned as two essential pillars for the communication.

#### 4.1.5. Step 5: Taking Decisions

The implementation of mutual goals and actions is literally described as a step in decision-making and the fifth step in the decision-making model. Actions **other than euthanasia** (5A) range from treating and re-visiting to separating the pig in hospital pens. Several reports also emphasized the importance of **caring for pigs** (5B). This begins before the pigs’ health deteriorates, for example, by separating piglets by size, or assessing stress and inferiority in a given group. Other actions include providing comfortable bedding, providing easily accessible food and water, and ensuring targeted, frequent monitoring of the pigs’ condition.

#### 4.1.6. Step 6: Evaluate the Outcome

The sixth step of the process is the evaluation of previous decisions. In some cases, the **development of clinical signs** (6A) is to be evaluated within a specific time frame: “I would say, re-assess the rearing site an hour later”, (A:53). Participants describe that they re-evaluate their decisions and thoughts until they **feel certain** (6B). “There were situations when I was ready to kill the pig and then, I thought—ok, you get another day- because I was a bit unsure then”, (B:30). Moreover, the evaluation of treatment outcomes is an important consideration, specifically with respect to treatment success.

#### 4.1.7. Step 7: Reflect on the Process

When asked how decision-making can be improved, several interviewees advocate reflecting upon timely euthanasia decisions. In terms of self-awareness (7C), participants recognize their own limits regarding diagnostic processes. They assess if important information is missing or if they have experiences to judge a case. In terms of self-regulation (7A) and self-efficacy (7F), participants reconsider their motivation, and expresses emotional regulation when reporting that the act of killing is demanding, but the right thing to do. In several reports, interviewees show that they self-evaluate (7B) their considerations and self-analyze (7G) whether their own performance corresponds to their own goals (Box 7).

Box 7 **Reflect on the Process I**

“Sometimes I wonder, what would happen if we had an unannounced control and someone else would stand in front of the animal. Could I explain to this person that this animal has a chance? Could I sell this to someone else?” ^(A:77)^

“I guess it would be correct to provide medicine in the case of pain. This is done far too rarely, we do this way to rarely as well.” ^(A:79)^

When reconsidering decisions and actions, participants express their self-esteem (7E) by believing in themselves and their own reasoning. Similarly, participants express self-confidence (7D) by expressing personal opinions on difficult topics and sometimes correcting the interviewer’s paraphrases (Box 8).

Box 8 **Reflect on the Process II**

“I don’t want to sound arrogant, but if I, with my professional background and experience, cannot make a correct decision, who can?” ^(T5:1)^

“Well, you say there is no right and false but I would sure say that there are boundary situations or states that are untenable.” ^(B:60)^

Overall, most of the reports are suggestions *on* actions, and in some cases, also reflections *for* action (Box 9).

Box 9 **Reflect on the Actions**

“That is a point, I would think that a documentation enhances one’s own reflection”? ^(B:30)^

“We have to elaborate how we can transfer that in communication.” ^(TA:34)^

“I guess, I have to go to another farm and watch how pithing is done correctly.” ^(B:30)^

The reflection that was stimulated by the interview was explicitly praised by an interviewee: to improve decision-making, “you ask exactly the questions you are ask me, and that is important to get a clear line”, (TA:36).

In the previous sections, seven steps of reasoning about euthanasia-worthy conditions of pigs were presented. A further analysis of the data examined the nature of the participants’ reasoning.

### 4.2. Reasoning and Learning During the Process

In the reported decision-making processes regarding euthanasia, there are indications of intuitive and analytical thinking.

#### 4.2.1. Intuitive Reasoning

Participants report that reasoning relies on the subjective impression about the animal of concern and that decision-making is not a matter of a cognitive checklist. They emphasize that the reasoning is implicit. Therefore, verbalization of responses is difficult (Box 10).

Box 10 **Reflect on the Actions**

“It is difficult. Probably I would have to think a bit longer about this, but it is generally difficult to verbalize an answer, about what you see in detail, this overall impression.” ^(B:51)^

“It is always a bit difficult to describe that.” ^(TA:71)^

“I think we are doing well, but we haven’t been able to communicate well yet.” ^(TC:22)^

In several cases, participants relied on their gut feeling, and one participant expressed concerns that his argument was not sufficiently convincing: “For certain diseased pigs you have, it is dull now when I am saying—a feeling- this is of course wrong, because—a feeling- what can you do with that in science”, (B:48). The expression of a “personal feeling” was not a single incident, but was reported in several cases. In only a few cases, participants grounded their decision on the basis of experience and experimental learning: “This is something that you can practice, that has to be repeated, and then it will work out”, (TC:49).

Another mentioned strategy is judging upon heuristics. Participants either define shortcut rules for decision-making or treatment. Additionally, participants express a rule-in rule-out logic of thought (i.e., a yes/no checklist) (Box 11).

Box 11 **Heuristics and Short-Cut Rules for Decision-Making**

“3-day-rule” ^L16:1^

“The first thing is, 99% of the animals always get a chance. That is a rule of our practice.” ^(TA:51)^

“Treatment successful after 1–2 days—yes/no? Follow-up treatment successful—yes/no?” ^(L21:1)^

The presented logics support the assumption that rapid judgment processes occur when evaluating euthanasia-worthy states. Further reports indicated that analytical reasoning plays a role in euthanasia decision-making as well.

#### 4.2.2. Analytical Reasoning

Participants who addressed the topic of improving decision-making, emphasized that it involves cognitive effort, attention, and time to decide about the further course of a pig. In this context, one veterinarian shared his experience of spending a day meeting with an entire farm team and discussing nothing but euthanasia of compromised pigs to improve the decision-making skills (TA:36). Another participant outlined that guidelines and shortcut decisions are handy but tricky. The decision about euthanasia is not a “black and white” decision; it cannot be made according to a traffic light system.

Summarizing the Section 4 on the logics of thought, the reports are dominated by decisions made through quick reasoning. However, it was emphasized that the euthanasia decision-making may require more thorough consideration.

## 5. Discussion

The goal of this article was to explore how the perspective of clinical reasoning enhances understanding of decision-making processes about euthanasia of a compromised pig. In particular, the questions assessed whether typical steps of clinical reasoning can be identified, and how reported symptoms interdepend throughout reasoning. The discussion elaborates on the quality of collected data in a first step. Keeping limitations in mind, the second step is to discuss the main implications of findings for the training of euthanasia decision-making and further research.

The data was collected from an online survey, which participants could recruit themselves for, as well as from in-depth interviews, where persons were purposefully invited. The eligibility of participants was based on long-term experience with pigs and experience in deciding on and/or performing euthanasia of a compromised pig. In this regard, the participants are considered experts in the field, but they do not represent any person involved in euthanasia of compromised pigs in Germany and abroad. To enhance the quality of data, a well-structured, mixed-method analysis was conducted. It led to going back and forth in data and checking how inductively defined codes compare with the deductive approach to enhance the explication of the content analysis. Results were furthermore discussed within the author group that is concerned with timely euthanasia of compromised pigs for more than ten years. Several steps are thus taken to generate a solid analysis despite the fact that limitations of scope (number of participants), representativeness (interest of participants in the topic), understanding and inference (interpretation of reports) need to be considered when generalizing upon results.

Regarding the question of whether typical steps of clinical reasoning can be identified, the results of the analysis showed that all steps outlined in the concept of Agne et al. and its related studies are represented [10,11,16]. Reasoning about euthanasia begins with (re-)identifying the pig of concern and collecting data about the patient. It proceeds with making sense of collected information and generating clinical and contextual arguments. Subsequently, a decision is made, re-evaluated, and reflected upon. Decision-makers also reflected their goodness and self-performance during the process. In this respect, euthanasia decision-making can be considered a clinical reasoning process.

Regarding the question of how reported symptoms interdepend throughout reasoning, the network analysis showed which symptoms are central to reasoning about euthanasia-worthy states. The findings emphasized that most of the symptoms reported for one euthanasia-worthy state of a specific disorder correspond with symptoms reported for another disorder. On the one hand, this finding is not surprising since most of the reported disorders were similar and therefore the clinical symptoms overlap (e.g., disorders of the locomotor tract have a similar set of clinical signs). On the other hand, symptoms of other disorders overlapped with those of the locomotor disorders as well, such as a suspected CNS infection, conditions of low-viability pigs, or conditions due to accidents. Only a few symptoms were associated with one disorder in particular, such as a rupture wound in the case of an umbilical hernia. (The authors emphasize that a rupture may or may not appear during the presence of a hernia. The appearance thus does not indicate timely euthanasia.) Due to this, we assume that the symptoms represented in our figures are important to define a euthanasia-worthy state. Moreover, they are more salient for decision-making than defining the specific diagnosis of a compromised pig.

From a theoretical perspective, this consideration suggests that evaluating a euthanasia-worthy condition can be described as a script rather than euthanasia being an act in the decision-making process. Whether euthanasia-worthy states are generic scripts or slots of specific illness scripts cannot be defined on the basis of this analysis. Future research needs to assess whether the network of symptoms can be consolidated. Another question is whether specific symptoms ensure timely euthanasia decisions in particular, and thus, perform as alarm links (i.e., symptoms that certain scripts share and activate for efficient reasoning [14] (p. 1180)). Overall, the results underscore that euthanasia is not just one consequence when assessing a pig’s condition. In this respect, clinical reasoning helps understand the euthanasia decision as a sequence of steps that activate reasoning scripts and symptom networks in the mind.

The central euthanasia-worthy symptoms were non-ambulatory states, taking up no food and water, and bad general health conditions. When including considerations about the identification of pain and suffering, they locate it in the midst of the network. The results indicate that pain and suffering are latent, yet central considerations in euthanasia decision-making. Participants tend to focus on the symptoms themselves rather than on the inference from the symptoms to pain and suffering unless asked. Since unnecessary pain and suffering constitutes a legal requirement for euthanasia in Germany, strengthening inference should be a future focus of training approaches.

Overall, the visualization enhances understanding about central and peripheral considerations during decision-making about euthanasia. The network of symptoms is one of the first representations in research that visualizes a structure of euthanasia-worthy states of compromised pigs. Future research needs to explore the interdependence of symptoms with respect to a broader set of primary diagnoses and farming environments.

Overall, the results emphasize how clinical reasoning enhances understanding about euthanasia decision-making: the approach allows synthesizing findings of previous studies into a logical order of thought. Especially the contextual arguments of this article correspond with findings in previous studies [1,6,7]. So far, however, the influencing factors have not been associated with a particular step of reasoning as outlined in this article. Since a logical order of thoughts can be decisive for the correct decision-making [14,18], we assume that training about typical reasoning steps may enhance euthanasia decision processes.

The results of the clinical reasoning approach also enhance understanding about specific challenges, such as predicting the chance of recovery. Figure 4 shows that recovery symptoms are underrepresented, despite the fact that interviewees were specifically asked about clinical signs signaling the recovery of a pig. Due to this, we assume that training and research need to improve related knowledge. This conclusion is supported by previous studies where research on disease trajectories has been advocated, which includes assessing signs indicating recovery of a compromised pig [2,5].

Another challenge to timely euthanasia is the insecurity of decision-makers to define a clear diagnosis. Results of this article suggest that euthanasia decisions may be well founded regardless of knowledge of the specific diagnosis, as long as typical symptoms for a euthanasia-worthy state are perceived early. This argument is intended to emphasize that the lack of a specific diagnosis for a compromised pig is no reason to delay euthanasia. Avoiding unnecessary pain or suffering, as represented by the symptom network, is and remains a central concern for timely euthanasia. However, the argument that a specific diagnosis is not required for a decision does not imply that biomedical knowledge (about disease, etiology, pathology, etc.) is unimportant for decision-making. From a theoretical lens, this knowledge is essential to generate cognitive pathways in reasoning processes [14]. To impart biomedical knowledge in this respect, it is essential to augment awareness of how symptoms and information can overlap across diseases. This overlap can be indicative of euthanasia-worthy states, necessitating immediate action.

Using visualization to convey knowledge about symptoms that signal a condition worthy of euthanasia can help decision-makers understand and challenge their actual reasoning. By discussing these thought processes, underlying unconscious thought processes and weaknesses can be identified, such as missing connections between symptoms or diagnoses, or heuristics and shortcuts that may need to be revised. Recognizing these limitations can support more effective decision-making.

**Figure 5:**
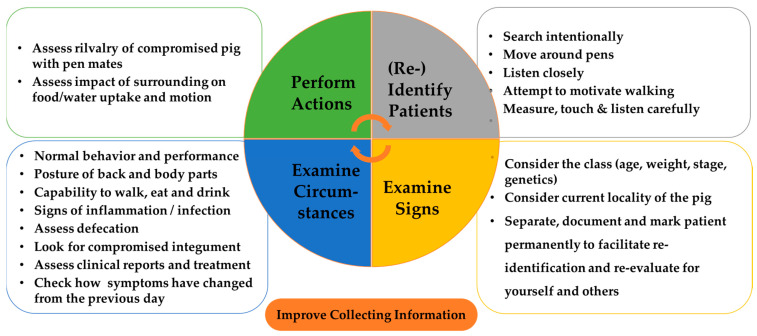
Raising awareness on how to improve collecting data about compromised pigs.

While Figure 1, Figure 2 and Figure 3 can be used to visualize symptom networks, Figure 5 summarizes key considerations for identifying and collecting data about compromised pigs, thereby enhancing the initial steps of a reasoning process.

**Figure 6:**
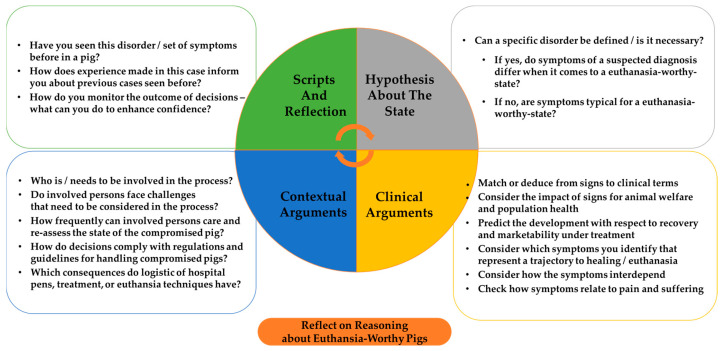
Reflect on reasoning about euthanasia-worthy pigs.

To highlight the implications for training reasoning processes, Figure 6 can be used in practice as well. It serves to elaborate on the reasoning process, allowing for the identification of which step was stressed or neglected during considerations. While Figure 5 and Figure 6 do not provide a definitive guide for determining when a compromised pig requires euthanasia, nor do they resolve the complexities of uncertain decision-making, they are intended to facilitate conscious reflection on the reasoning process.

In all cases of reasoning, whether driven by a negative or positive gut feeling, or an analytical re-evaluation of cases, engaging in reflective activities can help prevent the manifestation of insufficient cognitive pathways. By promoting self-awareness and introspection, these figures can aid in the development of more informed and effective decision-making processes. To conclude with a paraphrase of an interviewee report: “It is logical that a wrong decision is made sometimes. It is important however, that this isn’t made repeatedly, due to a systematical error”, (B:30).

## 6. Conclusions

For some stages of diseases or injuries of a pig, curing is impossible, and caretakers or veterinarians have to decide when the right time point has come to avoid unnecessary suffering and pain. To ensure timely euthanasia, training approaches have focused on the question of what subject knowledge needs to be learned. In the analysis of this article, the focus has been shifted to analyze how decisions are made and how reasoning about euthanasia proceeded in survey and interview reports. Based on the perspective of clinical reasoning, the analysis developed a seven-stage model of the euthanasia decision-making that can be used in practice to reflect on decisions about compromised pigs. The analysis also generated a visualization of symptom networks representing clinical signs central to the assessment of euthanasia-worthy conditions. The results are intended to facilitate future research on this topic and improve training on euthanasia-worthy conditions of compromised pigs. The results of the analysis demonstrate that clinical reasoning represents a fruitful perspective for analyzing euthanasia decision-making processes and contributes to organizing steps of the process into a logical sequence. Future research will have to corroborate findings of this explorative article that will contribute to enhancing timely euthanasia for the welfare of pigs and the mental health of involved persons.

## Figures and Tables

**Table 1:** Activities representing clinical reasoning processes of steps three and four. (The table synthesizes a table in [11] (p. 764) and a figure in [16] (p. 517)).

Activity	Explanation
Problem representation	Summarize gathered facts into a concise problem representation as a basis for differential hypotheses/diagnoses, use medical terms
Review context	Consider gathered facts regarding the examination of the environment
Problem identification	Synthesize facts and inferences to make a definitive diagnosis of the patient’s problem
Recall knowledge	Be aware of/recall previous exemplars, illness scripts, prototypes and used semantic qualifiers
Interpretation	Understand symptoms and cues of the case
Discrimination	Distinguish relevant from irrelevant information, define missing cues
Relating	Define patterns and clusters
Inferring	Make deductions or logical flows from considerations
Matching	Match findings with previous situations
Predicting	Predict an outcome

**Table 2:** Activities representing reflective practices of step eight. (The content is adapted from a table showing skills used in reflective practice in [10] (p. 19)).

Activity	Explanation
Self-analysis	A person reflects on whether the performance led to reach goals for the encounter
Self-awareness	Handling criticism and/or recognizing own limits
Self-confidence	Speak in awkward situations
Self-efficacy	Dedicate extra time to self-reflect and enhance ongoing and future encounters
Self-evaluation	Check if a decision needs to be adjusted
Self-regulation	Control the behavior/expressions of the affective state

**Table 3:** Steps completed when reasoning about euthanasia.

No.	Step	Activities (Subordinate Steps of Consideration)
1	Identify the patient	A Early Identification
2	Gathering data	A Specific actions
		B Relevant outcomes B1: Patient data B2: General examination goals B3: Abnormal symptoms
		C Patient circumstances
3	Analyzing data and interpreting steps	A Suspected hypotheses (diagnoses or hypothesis about the state of health) A1: Disorder/diagnosis vs. euthanasia-worthy states A2: Specificity of hypotheses
		B Clinical arguments for a hypothesis B1: Matching B2: Inferring B3: Relating B4: Predicting B5: Discriminating
		C Contextual arguments for a hypothesis C1: Characteristics of involved persons C2: Institutional structures C3: Farm and local equipment C4: “Individual case” theme
		D Recalled scripts D1: Specific illness scripts D2: Semantic qualifiers
4	Establish mutual goals	A Population health consequence
		B Farmer–veterinarian consultation and communication
5	Taking actions	A Other actions than euthanasia
		B Role of caring for pigs
6	Evaluate the outcome	A Development of clinical signs
		B Role of uncertainty
7	Reflect on the process	A Self-regulation
		B Self-evaluation/monitoring
		C Self-awareness
		D Self-confidence
		E Self-esteem
		F Self-efficacy
		G Self-analysis

## Data Availability

The data obtained were processed and evaluated anonymously and in compliance with the EU’s General Data Protection Regulation as well as with all data privacy regulations from Germany and the Federal State of Lower Saxony. The data are not publicly available due to ethical and privacy restrictions, i.e., any data transfer to interested persons is not allowed without a specific formal contract. This contract is available to qualified researchers and will include guarantees of the obligation to maintain data confidentiality in accordance with the provisions of the German data protection law. Currently, there is no data access committee or another body that could be contacted for the data. However, for this purpose, a committee will be founded. This future committee will consist of the authors as well as members of the University of Veterinary Medicine Hannover and members of the funding institution. Interested cooperative partners who are able to sign a contract as described above may contact the corresponding author.

## Appendix A

The interview questions are outlined in the following (translation of the authors):

- How long have you been working with pigs?
- When did you complete an agricultural vocational qualification (farmer)/since when do you hold a veterinary license (veterinarians)?
- Have you completed any further education?
- What is your position on farm (farmer)/in practice (veterinarians)
- What is the average size of the herds on the farm that you care for?
- Is “euthanasia of compromised pigs” a topic that is of general concern for you?
- Is there anything about euthanasia of compromised pigs that has attracted particular attention among your colleagues?
- For farmers only:
  ◦ Is it one of your tasks to decide about euthanasia of a pig?
  ◦ Which technique do you use for euthanasia?
- When was the last time you euthanized a compromised pig?
- For veterinarians only
  ◦ Who is rather conducting euthanasia—farmers or veterinarians?
- In which cases of diseases or injuries do you often find yourself in a situation where you have to consider euthanasia?
  ◦ If it is a long list: In which of the mentioned cases do you find it particularly difficult to determine the right time-point for euthanasia?
  ◦ In addition for veterinarians: Are these cases particularly difficult for farmers, too?
- For each disorder/injury X asked repeatedly:
  ◦ What of this pig attracts your attention on day one? Or: in which state is a pig presented to you the first time?
  ◦ How do you determine for a pig with X whether the state of a pig is improving/develops to recovery?
  ◦ How do you elaborate for a pig with X whether the state of a pig is worsening/develops to euthanasia?
    ▪ Answer to requests about the meaning of “elaborating”: what comes into mind as considerations and observations
- How do you identify that a pig feels pain?
- How do you identify that a pig is suffering?
- What kind of information or topics would have to be addressed in a guideline to improve the situation
- Is there anything left that you want to talk about, any question or issue left open?
